# Supplementary material for: A Computational Framework for Prediction and Analysis of Cancer Signaling Dynamics from RNA Sequencing Data—Application to the ErbB Receptor Signaling Pathway
Source: Cancers (Basel). 2020 Oct 7;12(10):2878. doi: 10.3390/cancers12102878 (PMC7650612; doi:10.3390/cancers12102878)
Supplement: Supplementary file 1 [file cancers-12-02878-s001.zip › SupplementaryMaterial0928/MethodS1.docx]

"""Integrated model

- Birtwistle et al., Mol. Syst. Biol. (2007)

- Nakakuki et al., Cell (2010)

Parameter

---------

t: time

y: state variable

x: constant parameter

Return

------

dydt: time derivative of y

Description of variable names

-----------------------------

1. Birtwistle et al.'s model (v[1] ~ v[93])

- G: Grb2.

- sigmaG: Grb2-containing species in which the Grb2 SH2 domain is bound to

tyrosine-phosphorylated receptor dimer (EijP) or to tyrosine-phosphorylated Shc (SP),

and both Grb2 SH3 domains are unbound.

- S: Shc.

- sigmaS: Shc-containing species in which the Shc SH2 domain is bound to

tyrosine-phosphorylated receptor dimer (EijP) or to membrane-localized,

tyrosine-phosphorylated GAB1 (AP), and Shc is unphosphorylated.

- I: PI-3K.

- sigmaI: PI-3K-containing species in which PI-3K is bound to

tyrosine-phosphorylated receptor dimer (EijP) or to membrane-localized,

tyrosine-phosphorylated GAB1 (AP).

- R: RasGAP.

- sigmaR: RasGAP-containing species in which RasGAP is bound to

tyrosine-phosphorylated receptor dimer (EijP) or to membrane-localized,

tyrosine-phosphorylated GAB1 (AP), but is not phosphorylated.

- T: PTP-1B.

- sigmaT: PTP-1B-containing species in which PTP-1B is bound to

tyrosine-phosphorylated receptor dimer (EijP) or to membrane-localized,

tyrosine-phosphorylated GAB1 (AP).

- A: GAB1.

- sigmaA: Gab1-containing species in which the GAB1 PH domain is bound to

PIP3 or the PRD is bound to Grb2, and GAB1 is unphosphorylated.

- O: SOS.

- sigmaO: SOS-containing species that are bound to

a membrane- localized N-terminal SH3 domain of Grb2.

2. Nakakuki et al.'s model (v[94] ~ v[156])

- ***c: cytoplasmic ***.

- ***n: nuclear ***.

"""

# extracellular volume to cytoplasmic volume ratio

VeVc = 33.3

# fraction definitions

if y[V.sigmaS] + y[V.sigmaSP] + y[V.sigmaSP_G] > 0.0:

fsigmaS = y[V.sigmaS]/(y[V.sigmaS] + y[V.sigmaSP] + y[V.sigmaSP_G])

else:

fsigmaS = 0.0

if y[V.sigmaG] + y[V.sigmaG_A] + y[V.sigmaG_O] + y[V.A_sigmaG_O] > 0.0:

fsigmaG = y[V.sigmaG]/(y[V.sigmaG] + y[V.sigmaG_A] + y[V.sigmaG_O] + y[V.A_sigmaG_O])

else:

fsigmaG = 0.0

if y[V.sigmaA] + y[V.sigmaAP] + y[V.sigmaAP_S] + y[V.sigmaAP_R] + y[V.sigmaAP_I] + y[V.sigmaAP_T] > 0.0:

fsigmaA = y[V.sigmaA]/(y[V.sigmaA] + y[V.sigmaAP] + y[V.sigmaAP_S] + y[V.sigmaAP_R] + y[V.sigmaAP_I] + y[V.sigmaAP_T])

else:

fsigmaA = 0.0

if y[V.sigmaR] + y[V.sigmaRP] > 0.0:

fsigmaR = y[V.sigmaR]/(y[V.sigmaR] + y[V.sigmaRP])

else:

fsigmaR = 0.0

sigmaEP = y[V.E11P] + y[V.E12P] + y[V.E23P] + y[V.E24P] + y[V.E34P] + y[V.E44P] + y[V.E13P] + y[V.E14P]

if sigmaEP > 0.0:

f11 = y[V.E11P]/sigmaEP

else:

f11 = 0.0

v = {}

# Birtwistle et al., MSB (2007)

v[1] = (x[C.kon1]*y[V.E]*y[V.E1] - x[C.EGF_off]*y[V.E_E1])

v[2] = (x[C.kon2]*y[V.H]*y[V.E3] - x[C.HRGoff_3]*y[V.H_E3])

v[3] = (x[C.kon3]*y[V.H]*y[V.E4] - x[C.HRGoff_4]*y[V.H_E4])

v[4] = (x[C.kon4]*y[V.E_E1]*y[V.E_E1] - x[C.koff4]*y[V.E11])

v[5] = (x[C.kon5]*y[V.E_E1]*y[V.E2] - x[C.koff5]*y[V.E12])

v[6] = (x[C.kon6]*y[V.H_E3]*y[V.E2] - x[C.koff6]*y[V.E23])

v[7] = (x[C.kon7]*y[V.H_E3]*y[V.H_E4] - x[C.koff7]*y[V.E34])

v[8] = (x[C.kon8]*y[V.H_E4]*y[V.E2] - x[C.koff8]*y[V.E24])

v[9] = (x[C.kon9]*y[V.H_E4]*y[V.H_E4] - x[C.koff9]*y[V.E44])

v[10] = (x[C.kf10]*y[V.E11] - x[C.VmaxPY]*y[V.E11P]/(x[C.KmPY] + y[V.E11P]) - x[C.kPTP10]*y[V.sigmaT]*y[V.E11P])

v[11] = (x[C.kf11]*y[V.E12] - x[C.VmaxPY]*y[V.E12P]/(x[C.KmPY] + y[V.E12P]) - x[C.kPTP11]*y[V.sigmaT]*y[V.E12P])

v[12] = (x[C.kf12]*y[V.E23] - x[C.VmaxPY]*y[V.E23P]/(x[C.KmPY] + y[V.E23P]) - x[C.kPTP12]*y[V.sigmaT]*y[V.E23P])

v[13] = (x[C.kf13]*y[V.E34] - x[C.VmaxPY]*y[V.E34P]/(x[C.KmPY] + y[V.E34P]) - x[C.kPTP13]*y[V.sigmaT]*y[V.E34P])

v[14] = (x[C.kf14]*y[V.E24] - x[C.VmaxPY]*y[V.E24P]/(x[C.KmPY] + y[V.E24P]) - x[C.kPTP14]*y[V.sigmaT]*y[V.E24P])

v[15] = (x[C.kf15]*y[V.E44] - x[C.VmaxPY]*y[V.E44P]/(x[C.KmPY] + y[V.E44P]) - x[C.kPTP15]*y[V.sigmaT]*y[V.E44P])

v[16] = (4*x[C.kon16]*y[V.E11P]*y[V.G] - x[C.koff16]*fsigmaG*y[V.E11G])

v[17] = (8*x[C.kon17]*y[V.E11P]*y[V.S] - x[C.koff17]*fsigmaS*y[V.E11S])

v[18] = (2*x[C.kon18]*y[V.E11P]*y[V.R] - x[C.koff18]*fsigmaR*y[V.E11R])

v[19] = (3*x[C.kon19]*y[V.E12P]*y[V.G] - x[C.koff19]*fsigmaG*y[V.E12G])

v[20] = (6*x[C.kon20]*y[V.E12P]*y[V.S] - x[C.koff20]*fsigmaS*y[V.E12S])

v[21] = (2*x[C.kon21]*y[V.E12P]*y[V.R] - x[C.koff21]*fsigmaR*y[V.E12R])

v[22] = (3*x[C.kon22]*y[V.E23P]*y[V.G] - x[C.koff22]*fsigmaG*y[V.E23G])

v[23] = (3*x[C.kon23]*y[V.E23P]*y[V.S] - x[C.koff23]*fsigmaS*y[V.E23S])

v[24] = (3*x[C.kon24]*y[V.E23P]*y[V.I] - x[C.koff24]*y[V.E23I])

v[25] = (2*x[C.kon25]*y[V.E23P]*y[V.R] - x[C.koff25]*fsigmaR*y[V.E23R])

v[26] = (4*x[C.kon26]*y[V.E34P]*y[V.G] - x[C.koff26]*fsigmaG*y[V.E34G])

v[27] = (3*x[C.kon27]*y[V.E34P]*y[V.S] - x[C.koff27]*fsigmaS*y[V.E34S])

v[28] = (4*x[C.kon28]*y[V.E34P]*y[V.I] - x[C.koff28]*y[V.E34I])

v[29] = (2*x[C.kon29]*y[V.E34P]*y[V.R] - x[C.koff29]*fsigmaR*y[V.E34R])

v[30] = (3*x[C.kon30]*y[V.E24P]*y[V.G] - x[C.koff30]*fsigmaG*y[V.E24G])

v[31] = (4*x[C.kon31]*y[V.E24P]*y[V.S] - x[C.koff31]*fsigmaS*y[V.E24S])

v[32] = (1*x[C.kon32]*y[V.E24P]*y[V.I] - x[C.koff32]*y[V.E24I])

v[33] = (2*x[C.kon33]*y[V.E24P]*y[V.R] - x[C.koff33]*fsigmaR*y[V.E24R])

v[34] = (4*x[C.kon34]*y[V.E44P]*y[V.G] - x[C.koff34]*fsigmaG*y[V.E44G])

v[35] = (4*x[C.kon35]*y[V.E44P]*y[V.S] - x[C.koff35]*fsigmaS*y[V.E44S])

v[36] = (2*x[C.kon36]*y[V.E44P]*y[V.I] - x[C.koff36]*y[V.E44I])

v[37] = (2*x[C.kon37]*y[V.E44P]*y[V.R] - x[C.koff37]*fsigmaR*y[V.E44R])

v[38] = (x[C.kf38]*y[V.sigmaS]*sigmaEP - x[C.VmaxPY]*y[V.sigmaSP]/(x[C.KmPY] + y[V.sigmaSP]) - x[C.kPTP38]*y[V.sigmaT]*y[V.sigmaSP])

v[39] = (x[C.kf39]*y[V.sigmaA]*sigmaEP - x[C.VmaxPY]*y[V.sigmaAP]/(x[C.KmPY] + y[V.sigmaAP]) - x[C.kPTP39]*y[V.sigmaT]*y[V.sigmaAP])

v[40] = (x[C.kon40]*y[V.sigmaG]*y[V.O] - x[C.koff40]*y[V.sigmaG_O])

v[41] = (x[C.kon41]*y[V.sigmaG]*y[V.A] - x[C.koff41]*y[V.sigmaG_A]*fsigmaA)

v[42] = (x[C.kon42]*y[V.sigmaSP]*y[V.G] - x[C.koff42]*y[V.sigmaSP_G]*fsigmaG)

v[43] = (3*x[C.kon43]*y[V.sigmaAP]*y[V.S] - x[C.koff43]*y[V.sigmaAP_S]*fsigmaS)

v[44] = (3*x[C.kon44]*y[V.sigmaAP]*y[V.I] - x[C.koff44]*y[V.sigmaAP_I])

v[45] = (2*x[C.kon45]*y[V.sigmaAP]*y[V.R] - x[C.koff45]*y[V.sigmaAP_R]*fsigmaR)

v[46] = (x[C.kon46]*y[V.P3]*y[V.A] - x[C.koff46]*y[V.P3_A]*fsigmaA)

v[47] = (x[C.kf47]*y[V.P3]*y[V.Akt]/(x[C.Kmf47] + y[V.Akt]) - x[C.Vmaxr47]*y[V.Aktstar]/(x[C.Kmr47] + y[V.Aktstar]))

v[48] = (x[C.kf48]*(1 - y[V.fint]*f11)*y[V.sigmaI]*y[V.P2]/(x[C.Kmf48] + y[V.P2]) - 3*x[C.PTEN]*y[V.P3]/(x[C.Kmr48] + y[V.P3]))

v[49] = (x[C.kf49]*y[V.sigmaO]*y[V.RsD]/(x[C.Kmf49] + y[V.RsD]) - x[C.kr49]*y[V.sigmaR]*y[V.RsT]/(x[C.Kmr49] + y[V.RsT]) - x[C.kr49b]*y[V.sigmaRP]*y[V.RsT]/(x[C.Kmr49b] + y[V.RsT]) - x[C.kcon49]*y[V.RsT])

v[50] = (x[C.kf50]*y[V.sigmaR]*sigmaEP - x[C.VmaxPY]*y[V.sigmaRP]/(x[C.KmPY] + y[V.sigmaRP]) - x[C.kPTP50]*y[V.sigmaT]*y[V.sigmaRP])

v[51] = (x[C.kf51]*y[V.RsT]*y[V.Raf]/(x[C.Kmf51] + y[V.Raf]) - x[C.Vmaxr51]*y[V.Rafstar]/(x[C.Kmrb51] + y[V.Rafstar]))

v[52] = (x[C.kf52]*y[V.Rafstar]*y[V.MEK]/(x[C.Kmf52] + y[V.MEK]) - x[C.Vmaxr52]*y[V.ppMEKc]/(x[C.Kmr52] + y[V.ppMEKc]))

# v[53]: No reaction

v[54] = (x[C.kf54]*y[V.O]*y[V.ppERKc]/(x[C.Kmf54] + y[V.O]) - x[C.Vmaxr54]*y[V.OP]/(x[C.Kmr54] + y[V.OP]))

v[55] = (x[C.kf55]*y[V.A]*y[V.ppERKc]/(x[C.Kmf55] + y[V.A]) - x[C.Vmaxr55]*y[V.AP]/(x[C.Kmr55] + y[V.AP]))

# v[56]: No reaction

v[57] = (x[C.kon57]*y[V.P3_A]*y[V.G] - x[C.koff57]*y[V.sigmaA_G])

v[58] = (x[C.kon58]*y[V.sigmaA_G]*y[V.O] - x[C.koff58]*y[V.sigmaA_G_O])

v[59] = (x[C.kon59]*y[V.sigmaG_O]*y[V.A] - x[C.koff59]*y[V.A_sigmaG_O]*fsigmaA)

v[60] = (x[C.kon60]*y[V.sigmaG_A]*y[V.O] - x[C.koff60]*y[V.A_sigmaG_O])

v[61] = (x[C.kon61]*y[V.H_E3]*y[V.E_E1] - x[C.koff61]*y[V.E13])

v[62] = (x[C.kon62]*y[V.H_E4]*y[V.E_E1] - x[C.koff62]*y[V.E14])

v[63] = (x[C.kf63]*y[V.E13] - x[C.VmaxPY]*y[V.E13P]/(x[C.KmPY] + y[V.E13P]) - x[C.kPTP63]*y[V.sigmaT]*y[V.E13P])

v[64] = (x[C.kf64]*y[V.E14] - x[C.VmaxPY]*y[V.E14P]/(x[C.KmPY] + y[V.E14P]) - x[C.kPTP64]*y[V.sigmaT]*y[V.E14P])

v[65] = (4*x[C.kon65]*y[V.E13P]*y[V.G] - x[C.koff65]*fsigmaG*y[V.E13G])

v[66] = (5*x[C.kon66]*y[V.E13P]*y[V.S] - x[C.koff66]*fsigmaS*y[V.E13S])

v[67] = (3*x[C.kon67]*y[V.E13P]*y[V.I] - x[C.koff67]*y[V.E13I])

v[68] = (2*x[C.kon68]*y[V.E13P]*y[V.R] - x[C.koff68]*fsigmaR*y[V.E13R])

v[69] = (4*x[C.kon69]*y[V.E14P]*y[V.G] - x[C.koff69]*fsigmaG*y[V.E14G])

v[70] = (6*x[C.kon70]*y[V.E14P]*y[V.S] - x[C.koff70]*fsigmaS*y[V.E14S])

v[71] = (1*x[C.kon71]*y[V.E14P]*y[V.I] - x[C.koff71]*y[V.E14I])

v[72] = (2*x[C.kon72]*y[V.E14P]*y[V.R] - x[C.koff72]*fsigmaR*y[V.E14R])

v[73] = (4*x[C.kon73]*y[V.E11P]*y[V.T] - x[C.koff73]*y[V.E11T])

v[74] = (3*x[C.kon74]*y[V.E12P]*y[V.T] - x[C.koff74]*y[V.E12T])

v[75] = (2*x[C.kon75]*y[V.E23P]*y[V.T] - x[C.koff75]*y[V.E23T])

v[76] = (2*x[C.kon76]*y[V.E34P]*y[V.T] - x[C.koff76]*y[V.E34T])

v[77] = (2*x[C.kon77]*y[V.E24P]*y[V.T] - x[C.koff77]*y[V.E24T])

v[78] = (2*x[C.kon78]*y[V.E44P]*y[V.T] - x[C.koff78]*y[V.E44T])

v[79] = (3*x[C.kon79]*y[V.E13P]*y[V.T] - x[C.koff79]*y[V.E13T])

v[80] = (3*x[C.kon80]*y[V.E14P]*y[V.T] - x[C.koff80]*y[V.E14T])

v[81] = (x[C.kf81]*y[V.E1]*y[V.ppERKc]/(x[C.Kmf81] + y[V.E1]) - x[C.Vmaxr81]*y[V.E1_PT]/(x[C.Kmr81] + y[V.E1_PT]))

v[82] = (x[C.kf82]*y[V.E2]*y[V.ppERKc]/(x[C.Kmf82] + y[V.E2]) - x[C.Vmaxr82]*y[V.E2_PT]/(x[C.Kmr82] + y[V.E2_PT]))

v[83] = (x[C.kf83]*y[V.E4]*y[V.ppERKc]/(x[C.Kmf83] + y[V.E4]) - x[C.Vmaxr83]*y[V.E4_PT]/(x[C.Kmr83] + y[V.E4_PT]))

v[84] = (x[C.kf84]*y[V.E_E1]*y[V.ppERKc]/(x[C.Kmf84] + y[V.E_E1]) - x[C.Vmaxr84]*y[V.E_E1_PT]/(x[C.Kmr84] + y[V.E_E1_PT]))

v[85] = (x[C.kf85]*y[V.H_E4]*y[V.ppERKc]/(x[C.Kmf85] + y[V.H_E4]) - x[C.Vmaxr85]*y[V.H_E4_PT]/(x[C.Kmr85] + y[V.H_E4_PT]))

v[86] = (x[C.kon86]*y[V.E]*y[V.E1_PT] - x[C.EGF_off]*y[V.E_E1_PT])

v[87] = (x[C.kon87]*y[V.H]*y[V.E4_PT] - x[C.HRGoff_4]*y[V.H_E4_PT])

v[88] = (2*x[C.kon88]*y[V.sigmaAP]*y[V.T] - x[C.koff88]*y[V.sigmaAP_T])

# E11 degradation

v[89] = x[C.kdeg]*y[V.E11P]

v[90] = x[C.kdeg]*y[V.E11G]

v[91] = x[C.kdeg]*y[V.E11S]

v[92] = x[C.kdeg]*y[V.E11R]

v[93] = x[C.kdeg]*y[V.E11T]

# Nakakuki et al., Cell (2010)

v[94] = x[C.V1] * y[V.ppMEKc] * y[V.ERKc] / ( x[C.Km1] * (1 + y[V.pERKc] / x[C.Km2]) + y[V.ERKc] )

v[95] = x[C.V2] * y[V.ppMEKc] * y[V.pERKc] / ( x[C.Km2] * (1 + y[V.ERKc] / x[C.Km1]) + y[V.pERKc] )

v[96] = x[C.V3] * y[V.pERKc] / ( x[C.Km3] * (1 + y[V.ppERKc] / x[C.Km4]) + y[V.pERKc] )

v[97] = x[C.V4] * y[V.ppERKc] / ( x[C.Km4]* (1 + y[V.pERKc] / x[C.Km3]) + y[V.ppERKc] )

v[98] = x[C.V5] * y[V.pERKn] / ( x[C.Km5] * (1 + y[V.ppERKn] / x[C.Km6]) + y[V.pERKn] )

v[99] = x[C.V6] * y[V.ppERKn] / ( x[C.Km6] * (1 + y[V.pERKn] / x[C.Km5]) + y[V.ppERKn] )

v[100] = x[C.KimERK] * y[V.ERKc] - x[C.KexERK] * (x[C.Vn]/x[C.Vc]) * y[V.ERKn]

v[101] = x[C.KimpERK] * y[V.pERKc] - x[C.KexpERK] * (x[C.Vn]/x[C.Vc]) * y[V.pERKn]

v[102] = x[C.KimppERK] * y[V.ppERKc] - x[C.KexppERK] * (x[C.Vn]/x[C.Vc]) * y[V.ppERKn]

v[103] = x[C.V10] * y[V.ppERKn]**x[C.n10] / ( x[C.Km10]**x[C.n10] + y[V.ppERKn]**x[C.n10] )

v[104] = x[C.p11] * y[V.PreduspmRNAn]

v[105] = x[C.p12] * y[V.duspmRNAc]

v[106] = x[C.p13] * y[V.duspmRNAc]

v[107] = x[C.V14] * y[V.ppERKc] * y[V.DUSPc] / ( x[C.Km14] + y[V.DUSPc] )

v[108] = x[C.V15] * y[V.pDUSPc] / ( x[C.Km15] + y[V.pDUSPc] )

v[109] = x[C.p16] * y[V.DUSPc]

v[110] = x[C.p17] * y[V.pDUSPc]

v[111] = x[C.KimDUSP] * y[V.DUSPc] - x[C.KexDUSP] * (x[C.Vn]/x[C.Vc]) * y[V.DUSPn]

v[112] = x[C.KimpDUSP] * y[V.pDUSPc] - x[C.KexpDUSP] * (x[C.Vn]/x[C.Vc]) * y[V.pDUSPn]

v[113] = x[C.V20] * y[V.ppERKn] * y[V.DUSPn] / ( x[C.Km20] + y[V.DUSPn] )

v[114] = x[C.V21] * y[V.pDUSPn] / ( x[C.Km21] + y[V.pDUSPn] )

v[115] = x[C.p22] * y[V.DUSPn]

v[116] = x[C.p23] * y[V.pDUSPn]

v[117] = x[C.V24] * y[V.ppERKc] * y[V.RSKc] / ( x[C.Km24] + y[V.RSKc] )

v[118] = x[C.V25] * y[V.pRSKc] / ( x[C.Km25] + y[V.pRSKc] )

v[119] = x[C.KimRSK] * y[V.pRSKc] - x[C.KexRSK] * (x[C.Vn]/x[C.Vc]) * y[V.pRSKn]

v[120] = x[C.V27] * y[V.pRSKn] * y[V.CREBn] / ( x[C.Km27] + y[V.CREBn] )

v[121] = x[C.V28] * y[V.pCREBn] / ( x[C.Km28] + y[V.pCREBn] )

v[122] = x[C.V29] * y[V.ppERKn] * y[V.Elk1n] / ( x[C.Km29] + y[V.Elk1n] )

v[123] = x[C.V30] * y[V.pElk1n] / ( x[C.Km30] + y[V.pElk1n] )

v[124] = x[C.V31] * (y[V.pCREBn] * y[V.pElk1n])**x[C.n31] / ( x[C.Km31]**x[C.n31] + (y[V.pCREBn] * y[V.pElk1n])**x[C.n31] + (y[V.Fn] / x[C.KF31])**x[C.nF31] )

v[125] = x[C.p32] * y[V.PrecfosmRNAn]

v[126] = x[C.p33] * y[V.cfosmRNAc]

v[127] = x[C.p34] * y[V.cfosmRNAc]

v[128] = x[C.V35] * y[V.ppERKc] * y[V.cFOSc] / ( x[C.Km35] + y[V.cFOSc] )

v[129] = x[C.V36] * y[V.pRSKc] * y[V.cFOSc] / ( x[C.Km36] + y[V.cFOSc] )

v[130] = x[C.V37] * y[V.pcFOSc] / ( x[C.Km37] + y[V.pcFOSc] )

v[131] = x[C.p38] * y[V.cFOSc]

v[132] = x[C.p39] * y[V.pcFOSc]

v[133] = x[C.KimFOS] * y[V.cFOSc] - x[C.KexFOS] * (x[C.Vn]/x[C.Vc]) * y[V.cFOSn]

v[134] = x[C.KimpcFOS] * y[V.pcFOSc] - x[C.KexpcFOS] * (x[C.Vn]/x[C.Vc]) * y[V.pcFOSn]

v[135] = x[C.V42] * y[V.ppERKn] * y[V.cFOSn] / ( x[C.Km42] + y[V.cFOSn] )

v[136] = x[C.V43] * y[V.pRSKn] * y[V.cFOSn] / ( x[C.Km43] + y[V.cFOSn] )

v[137] = x[C.V44] * y[V.pcFOSn] / ( x[C.Km44] + y[V.pcFOSn] )

v[138] = x[C.p45] * y[V.cFOSn]

v[139] = x[C.p46] * y[V.pcFOSn]

v[140] = x[C.p47] * y[V.DUSPn] * y[V.ppERKn] - x[C.m47] * y[V.DUSPn_ppERKn]

v[141] = x[C.p48] * y[V.DUSPn_ppERKn]

v[142] = x[C.p49] * y[V.DUSPn] * y[V.pERKn] - x[C.m49] * y[V.DUSPn_pERKn]

v[143] = x[C.p50] * y[V.DUSPn_pERKn]

v[144] = x[C.p51] * y[V.DUSPn] * y[V.ERKn] - x[C.m51] * y[V.DUSPn_ERKn]

v[145] = x[C.p52] * y[V.pDUSPn] * y[V.ppERKn] - x[C.m52] * y[V.pDUSPn_ppERKn]

v[146] = x[C.p53] * y[V.pDUSPn_ppERKn]

v[147] = x[C.p54] * y[V.pDUSPn] * y[V.pERKn] - x[C.m54] * y[V.pDUSPn_pERKn]

v[148] = x[C.p55] * y[V.pDUSPn_pERKn]

v[149] = x[C.p56] * y[V.pDUSPn] * y[V.ERKn] - x[C.m56] * y[V.pDUSPn_ERKn]

v[150] = x[C.V57] * y[V.pcFOSn]**x[C.n57] / ( x[C.Km57]**x[C.n57] + y[V.pcFOSn]**x[C.n57] )

v[151] = x[C.p58] * y[V.PreFmRNAn]

v[152] = x[C.p59] * y[V.FmRNAc]

v[153] = x[C.p60] * y[V.FmRNAc]

v[154] = x[C.p61] * y[V.Fc]

v[155] = x[C.KimF] * y[V.Fc] - x[C.KexF] * (x[C.Vn]/x[C.Vc]) * y[V.Fn]

v[156] = x[C.p63] * y[V.Fn]

dydt[V.E1] = -v[1] - v[81]

dydt[V.E2] = -v[5] - v[6] - v[8] - v[82]

dydt[V.E3] = -v[2]

dydt[V.E4] = -v[3] - v[83]

dydt[V.E_E1] = v[1] - v[4] - v[4] - v[5] - v[61] - v[62] - v[84]

dydt[V.H_E3] = v[2] - v[6] - v[7] - v[61]

dydt[V.H_E4] = v[3] - v[7] - v[8] - v[9] - v[9] - v[62] - v[85]

dydt[V.E11] = v[4] - v[10]

dydt[V.E12] = v[5] - v[11]

dydt[V.E23] = v[6] - v[12]

dydt[V.E34] = v[7] - v[13]

dydt[V.E24] = v[8] - v[14]

dydt[V.E44] = v[9] - v[15]

dydt[V.E11P] = v[10] - v[16] - v[17] - v[18] - v[73] - v[89]

dydt[V.E12P] = v[11] - v[19] - v[20] - v[21] - v[74]

dydt[V.E23P] = v[12] - v[22] - v[23] - v[24] - v[25] - v[75]

dydt[V.E34P] = v[13] - v[26] - v[27] - v[28] - v[29] - v[76]

dydt[V.E24P] = v[14] - v[30] - v[31] - v[32] - v[33] - v[77]

dydt[V.E44P] = v[15] - v[34] - v[35] - v[36] - v[37] - v[78]

dydt[V.G] = -v[16] - v[19] - v[22] - v[26] - v[30] - v[34] - v[42] - v[57] - v[65] - v[69] + v[90]

dydt[V.S] = -v[17] - v[20] - v[23] - v[27] - v[31] - v[35] - v[43] - v[66] - v[70] + v[91]

dydt[V.I] = -v[24] - v[28] - v[32] - v[36] - v[44] - v[67] - v[71]

dydt[V.R] = -v[18] - v[21] - v[25] - v[29] - v[33] - v[37] - v[45] - v[68] - v[72] + v[92]

dydt[V.O] = -v[40] - v[54] - v[58] - v[60]

dydt[V.A] = -v[41] - v[46] - v[55] - v[59]

dydt[V.E11G] = v[16] - v[90]

dydt[V.E11S] = v[17] - v[91]

dydt[V.E11R] = v[18] - v[92]

dydt[V.E12G] = v[19]

dydt[V.E12S] = v[20]

dydt[V.E12R] = v[21]

dydt[V.E23G] = v[22]

dydt[V.E23S] = v[23]

dydt[V.E23I] = v[24]

dydt[V.E23R] = v[25]

dydt[V.E34G] = v[26]

dydt[V.E34S] = v[27]

dydt[V.E34I] = v[28]

dydt[V.E34R] = v[29]

dydt[V.E24G] = v[30]

dydt[V.E24S] = v[31]

dydt[V.E24I] = v[32]

dydt[V.E24R] = v[33]

dydt[V.E44G] = v[34]

dydt[V.E44S] = v[35]

dydt[V.E44I] = v[36]

dydt[V.E44R] = v[37]

dydt[V.sigmaG] = v[16] + v[19] + v[22] + v[26] + v[30] + v[34] - v[40] - v[41] + v[42] + v[65] + v[69] - v[90]

dydt[V.sigmaS] = v[17] + v[20] + v[23] + v[27] + v[31] + v[35] - v[38] + v[43] + v[66] + v[70] - v[91]

dydt[V.sigmaI] = v[24] + v[28] + v[32] + v[36] + v[44] + v[67] + v[71]

dydt[V.sigmaR] = v[18] + v[21] + v[25] + v[29] + v[33] + v[37] + v[45] - v[50] + v[68] + v[72] - v[92]

dydt[V.sigmaA] = -v[39] + v[41] + v[46] + v[59]

dydt[V.sigmaSP] = v[38] - v[42]

dydt[V.sigmaAP] = v[39] - v[43] - v[44] - v[45] - v[88]

dydt[V.sigmaG_O] = v[40] - v[59]

dydt[V.sigmaG_A] = v[41] - v[60]

dydt[V.sigmaSP_G] = v[42]

dydt[V.sigmaAP_S] = v[43]

dydt[V.sigmaAP_I] = v[44]

dydt[V.sigmaAP_R] = v[45]

dydt[V.P3_A] = v[46] - v[57]

dydt[V.P2] = -v[48]

dydt[V.P3] = -v[46] + v[48]

dydt[V.Akt] = -v[47]

dydt[V.RsD] = -v[49]

dydt[V.RsT] = v[49]

dydt[V.sigmaRP] = v[50]

dydt[V.Raf] = -v[51]

dydt[V.Rafstar] = v[51]

dydt[V.MEK] = -v[52]

dydt[V.ppMEKc] = v[52]

dydt[V.OP] = v[54]

dydt[V.AP] = v[55]

dydt[V.A_sigmaG_O] = v[59] + v[60]

dydt[V.sigmaA_G] = v[57] - v[58]

dydt[V.sigmaA_G_O] = v[58]

dydt[V.sigmaO] = v[40] + v[58] + v[60]

dydt[V.E13] = v[61] - v[63]

dydt[V.E14] = v[62] - v[64]

dydt[V.E13P] = v[63] - v[65] - v[66] - v[67] - v[68] - v[79]

dydt[V.E14P] = v[64] - v[69] - v[70] - v[71] - v[72] - v[80]

dydt[V.E13G] = v[65]

dydt[V.E13S] = v[66]

dydt[V.E13I] = v[67]

dydt[V.E13R] = v[68]

dydt[V.E14G] = v[69]

dydt[V.E14S] = v[70]

dydt[V.E14I] = v[71]

dydt[V.E14R] = v[72]

dydt[V.T] = -v[73] - v[74] - v[75] - v[76] - v[77] - v[78] - v[79] - v[80] - v[88] + v[93]

dydt[V.E11T] = v[73] - v[93]

dydt[V.E12T] = v[74]

dydt[V.E23T] = v[75]

dydt[V.E34T] = v[76]

dydt[V.E24T] = v[77]

dydt[V.E44T] = v[78]

dydt[V.E13T] = v[79]

dydt[V.E14T] = v[80]

dydt[V.sigmaT] = v[73] + v[74] + v[75] + v[76] + v[77] + v[78] + v[79] + v[80] + v[88] - v[93]

dydt[V.E1_PT] = v[81] - v[86]

dydt[V.E2_PT] = v[82]

dydt[V.E4_PT] = v[83] - v[87]

dydt[V.E_E1_PT] = v[84] + v[86]

dydt[V.H_E4_PT] = v[85] + v[87]

dydt[V.Aktstar] = v[47]

dydt[V.sigmaAP_T] = v[88]

dydt[V.E] = (-v[1] - v[86])/VeVc

dydt[V.H] = (-v[2] - v[3] - v[87])/VeVc

dydt[V.fint] = x[C.a98]*(-y[V.fint] + x[C.b98])

dydt[V.CREBn] = -v[120] + v[121]

dydt[V.pCREBn] = v[120] - v[121]

dydt[V.ERKc] = -v[94] + v[96] - v[100]

dydt[V.ERKn] = v[98] + v[100]*(x[C.Vc]/x[C.Vn]) + v[143] -v[144] + v[148] -v[149]

dydt[V.pERKc] = v[94] - v[95] -v[96] +v[97]-v[101]

dydt[V.pERKn] = -v[98] + v[99] + v[101]*(x[C.Vc]/x[C.Vn]) + v[141] - v[142] + v[146] - v[147]

dydt[V.ppERKc] = v[95] - v[97] - v[102]

dydt[V.ppERKn] = -v[99] + v[102]*(x[C.Vc]/x[C.Vn]) - v[140] - v[145]

dydt[V.Elk1n] = -v[122] + v[123]

dydt[V.pElk1n] = v[122] - v[123]

dydt[V.cFOSc] = v[127] - v[128] - v[129] + v[130] - v[131] - v[133]

dydt[V.cFOSn] = v[133]*(x[C.Vc]/x[C.Vn]) - v[135] - v[136] + v[137] - v[138]

dydt[V.pcFOSc] = v[128] + v[129] - v[130] - v[132] - v[134]

dydt[V.pcFOSn] = v[134]*(x[C.Vc]/x[C.Vn]) + v[135] + v[136] - v[137] - v[139]

dydt[V.DUSPc] = v[106] - v[107] + v[108] - v[109] - v[111]

dydt[V.DUSPn] = v[111]*(x[C.Vc]/x[C.Vn]) - v[113] + v[114] - v[115] - v[140] + v[141] - v[142] + v[143] - v[144]

dydt[V.pDUSPc] = v[107] - v[108] - v[110] - v[112]

dydt[V.pDUSPn] = v[112]*(x[C.Vc]/x[C.Vn]) + v[113] - v[114] - v[116] - v[145] + v[146] - v[147] + v[148] - v[149]

dydt[V.DUSPn_ERKn] = v[144]

dydt[V.DUSPn_pERKn] = v[142] - v[143]

dydt[V.DUSPn_ppERKn] = v[140] - v[141]

dydt[V.pDUSPn_ERKn] = v[149]

dydt[V.pDUSPn_pERKn] = v[147] - v[148]

dydt[V.pDUSPn_ppERKn] = v[145] - v[146]

dydt[V.RSKc] = -v[117] + v[118]

dydt[V.pRSKc] = v[117] - v[118] - v[119]

dydt[V.pRSKn] = v[119]*(x[C.Vc]/x[C.Vn])

dydt[V.PrecfosmRNAn] = v[124] - v[125]

dydt[V.PreduspmRNAn] = v[103] - v[104]

dydt[V.cfosmRNAc] = v[125]*(x[C.Vn]/x[C.Vc]) - v[126]

dydt[V.duspmRNAc] = v[104]*(x[C.Vn]/x[C.Vc]) - v[105]

dydt[V.Fc] = v[153] - v[154] - v[155]

dydt[V.Fn] = v[155]*(x[C.Vc]/x[C.Vn]) - v[156]

dydt[V.FmRNAc] = v[151]*(x[C.Vn]/x[C.Vc]) - v[152]

dydt[V.PreFmRNAn] = v[150] - v[151]
