# Supplementary material for: A Computational Framework for Prediction and Analysis of Cancer Signaling Dynamics from RNA Sequencing Data—Application to the ErbB Receptor Signaling Pathway
Source: Cancers (Basel). 2020 Oct 7;12(10):2878. doi: 10.3390/cancers12102878 (PMC7650612; doi:10.3390/cancers12102878)
Supplement: Supplementary file 1 [file cancers-12-02878-s001.zip › SupplementaryMaterial0928/FigureS2.pdf]

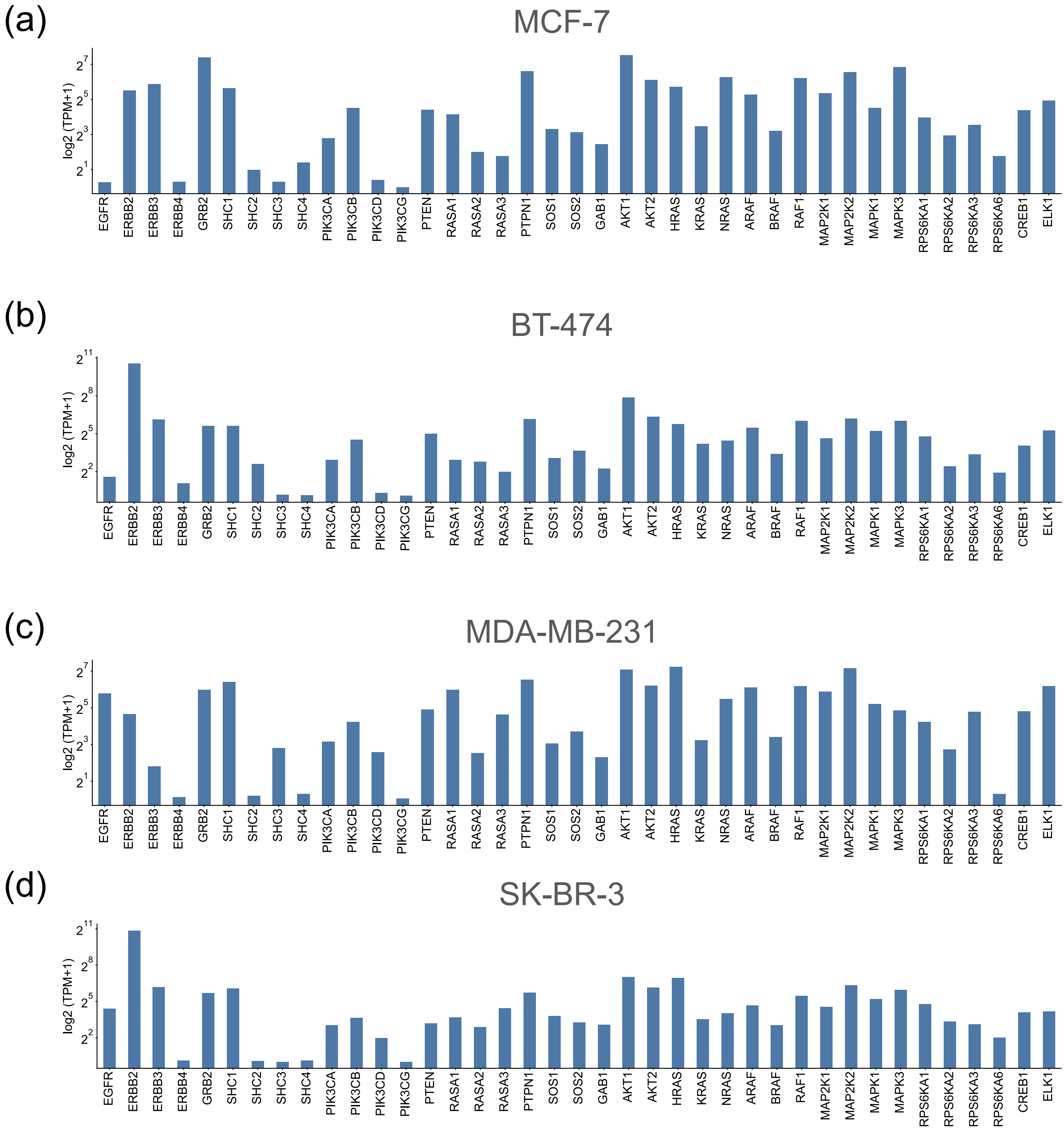

Figure S2. Input gene expression data used to train the mechanistic model and prediction. (**a**) MCF-7. (**b**) BT-474. (**c**) MDA-MB-231. (**d**) SK-BR-3.
