# Supplementary material for: A Computational Framework for Prediction and Analysis of Cancer Signaling Dynamics from RNA Sequencing Data—Application to the ErbB Receptor Signaling Pathway
Source: Cancers (Basel). 2020 Oct 7;12(10):2878. doi: 10.3390/cancers12102878 (PMC7650612; doi:10.3390/cancers12102878)
Supplement: Supplementary file 1 [file cancers-12-02878-s001.zip › SupplementaryMaterial0928/FigureS1.pdf]

(a)

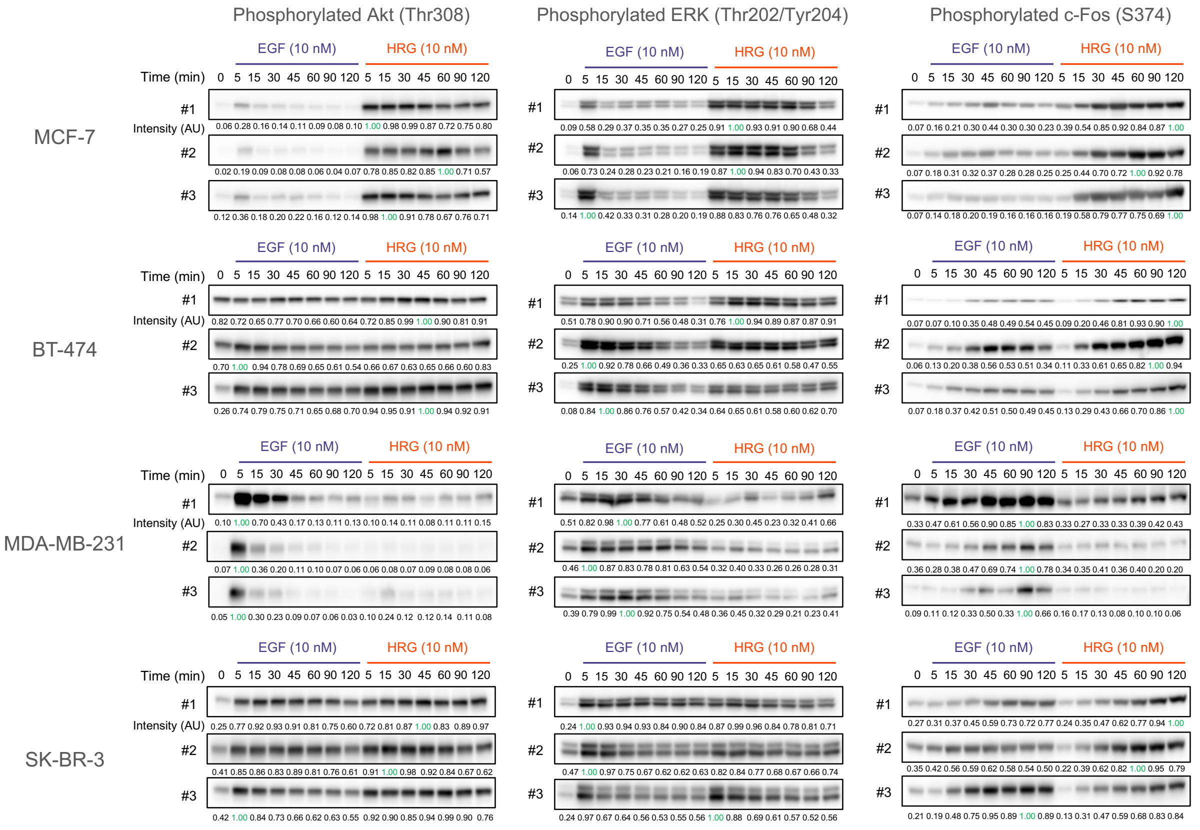

(b)

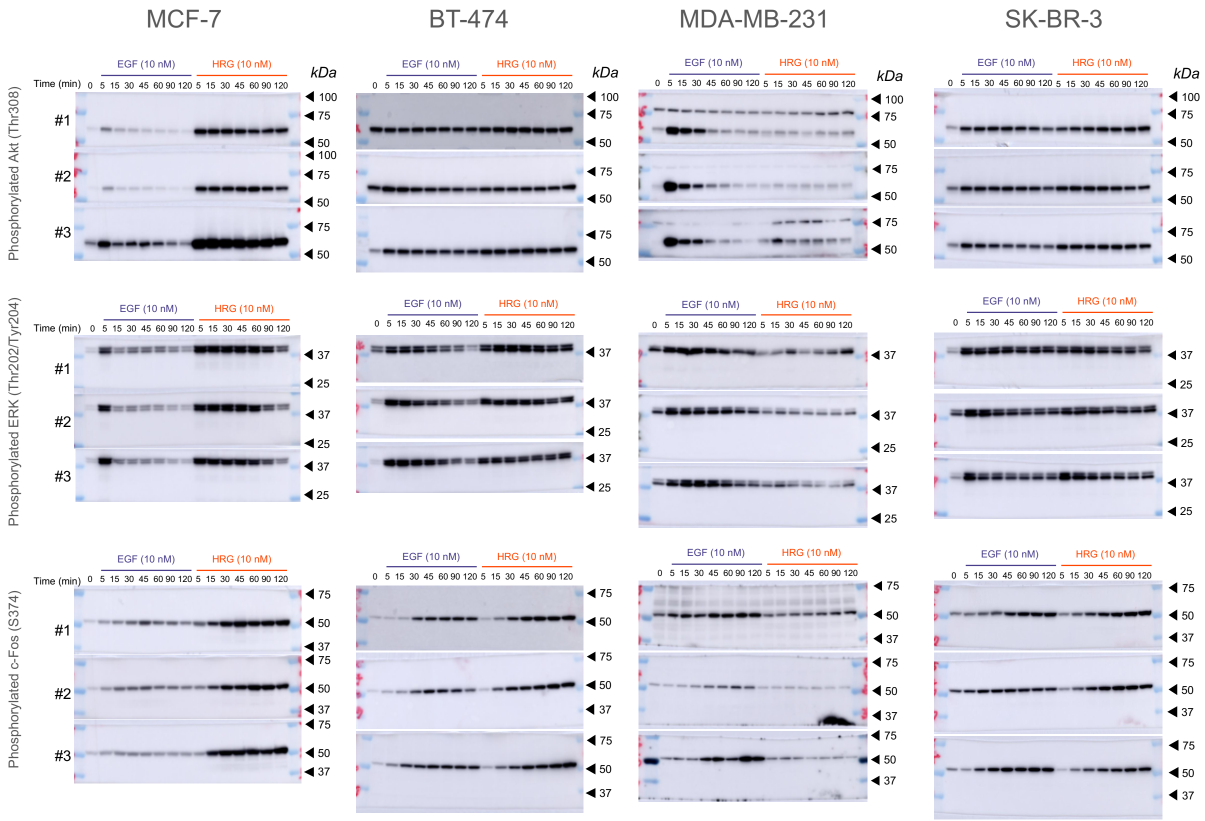

Figure S1. Experimental datasets used to train and validate the mechanistic model. (**a**) ErbB signals in breast cancer cell lines were exposed to 10 nM EGF and HRG. The abundance of phosphoproteins are assessed over 120 min. pAkt, pERK and pc-Fos were measured by western blotting. The numbers shown below each band indicate normalized signal intensities for the maximum values. (**b**) Raw immunoblot images. Molecular weights are indicated on the right.
